# Strain Elastography Fat-to-Lesion Index Is Associated with Mammography BI-RADS Grading, Biopsy, and Molecular Phenotype in Breast Cancer

**DOI:** 10.3390/bios14020094

**Published:** 2024-02-10

**Authors:** José Alfonso Cruz-Ramos, Mijaíl Irak Trapero-Corona, Ingrid Aurora Valencia-Hernández, Luz Amparo Gómez-Vargas, María Teresa Toranzo-Delgado, Karla Raquel Cano-Magaña, Emmanuel De la Mora-Jiménez, Gabriela del Carmen López-Armas

**Affiliations:** 1 Departamento de Clínicas Médicas, Instituto de Patología Infecciosa y Experimental, Centro Universitario de Ciencias de la Salud, Universidad de Guadalajara; Guadalajara 44340, Mexico; 2 Subdirección de Desarrollo Institucional, Instituto Jalisciense de Cancerología, Guadalajara 44280, Mexico; 3 Departamento de Ciencias Computacionales, Instituto Nacional de Astrofísica Óptica y Electrónica, San Andrés Cholula 72840, Mexico; 4 Laboratorio de Biomédica-Mecatrónica, Subdirección de Investigación y Extensión, Centro de Enseñanza Técnica Industrial Plantel Colomos, Guadalajara 44638, Mexico

**Keywords:** elastography, breast cancer, fat-to-lesion index

## Abstract

Breast cancer (BC) affects millions of women worldwide, causing over 500,000 deaths annually. It is the leading cause of cancer mortality in women, with 70% of deaths occurring in developing countries. Elastography, which evaluates tissue stiffness, is a promising real-time minimally invasive technique for BC diagnosis. This study assessed strain elastography (SE) and the fat-to-lesion (F/L) index for BC diagnosis. This prospective study included 216 women who underwent SE, ultrasound, mammography, and breast biopsy (108 malignant, 108 benign). Three expert radiologists performed imaging and biopsies. Mean F/L index was 3.70 ± 2.57 for benign biopsies and 18.10 ± 17.01 for malignant. We developed two predictive models: a logistic regression model with AUC 0.893, 79.63% sensitivity, 87.62% specificity, 86.9% positive predictive value (+PV), and 80.7% negative predictive value (−PV); and a neural network with AUC 0.902, 80.56% sensitivity, 88.57% specificity, 87.9% +PV, and 81.6% −PV. The optimal Youden F/L index cutoff was >5.76, with 84.26% sensitivity and specificity. The F/L index positively correlated with BI-RADS (Spearman’s r = 0.073, *p* < 0.001) and differed among molecular subtypes (Kruskal-Wallis, *p* = 0.002). SE complements mammography for BC diagnosis. With adequate predictive capacity, SE is fast, minimally invasive, and useful when mammography is contraindicated.

## 1. Introduction

Breast cancer (BC) is a major public health disease worldwide. GLOBOCAN reported a prevalence of 7,790,717 females with BC in 2020 [1]. Mammography, ultrasound, and magnetic resonance imaging (MRI) are commonly used for BC detection, applied based on specific characteristics of breast, such as density, microcalcifications, patient age, family history, and breast lesions at an early age (Table 1). The American College of Radiology recognizes elastography and digital breast tomosynthesis (DBT) as complementary techniques to reveal tissue components and deformity [2]. Elastography is a minimally invasive technique used to assess tissue stiffness via acoustic waves; it accounts for the anisotropic, viscous, and nonlinear mechanical behavior of tissues [3]. Elastography generates elastogram images which may then be used to quantify biomechanical properties like Young’s modulus, Poisson’s ratio, and strain [4]. Elastography consists of two categories: semi-quantitative or strain elastography (SE) and quantitative or shear−wave elastography (SWE) [5]. SE measures tissue elasticity through the compression of non-tumoral fat tissue and the elasticity of the lesion tissue as it deforms [6,7]. A radiologist compares both measurements for the fat-to-lesion index (F/L-index). On the other hand, SWE directly quantifies tissue elasticity by measuring the shear wave speed through tissue. It requires no manual compression. SWE sends low-energy acoustic pulses that displace tissue, inducing shear waves. By tracking the wave propagation speed, SWE calculates elasticity independently of applied pressure. Results are reported in meters per second (m/s) or kilopascals (kPa) [8,9].

Studies have evaluated the performance of elastography for SE and SWE. Youk et al. examined 257 breast masses with both SE and SWE. They acquired multiple ROIs over lesions and surrounding fat. Comparing ROI ratios provided a robust fat-to-lesion index for the identification of malignancies [10]. Similarly, Seo et al. evaluated 45 breast masses using combined SE and SWE. The merged elastography data had significantly higher diagnostic accuracy (*p* = 0.031) than those of either method alone [11]. Overall, the techniques are highly complementary.

Over the past 15 years, elastography (either SE or SWE) has become a valuable tool for differentiating between benign and malignant tumors in breast, as well as in other organs, including liver, prostate, and thyroid [12,13,14]. It is particularly useful for women under 40 years of age and those with dense breast tissue, where mammography has reduced sensitivity. Ultrasound is often used as a first-line screening modality in these groups, and elastography enhances its specificity [15,16]. The American College of Radiology recognizes elastography for its ability to characterize breast lesions [17]. Several studies have demonstrated the relationship between tissue elasticity and its association with BC [3,18,19].

This study aimed to determine the sensitivity, specificity, and optimal cut-off of the F/L index in the diagnosis of BC and benign tumors using SE. We also obtained BI-RADS, subtypes, stages, and grades in our study population.

**Table 1:** Different imaging techniques used for analyzing breast tissue.

Method	Principle	Invasiveness	Radiation	Detection	Operator-Dependent Bias	Time per Procedure	Cost	Availability	Specificity	Contraindications	References
Elastography	Through sound waves, tissue stiffness is measured	Minimally invasive	N/A	Useful in suspicious lesions	High variability	Fast procedure	Low cost	Wide availability	Moderate	Patients with conventional ultrasound difficulties	[5]
Mammography	Uses X-rays to obtain 2D images of the breast	Breast compression	Ionizing radiation	Useful to identify calcifications	High variability	Fast procedure	Moderate cost	Wide availability	Moderate to low	Not recommended in young or pregnant women	[20]
Magnetic resonance imaging (MRI)	Based on the application of magnetic fields and radio waves	Minimally invasive	Non-ionizing radiation	Useful to identify calcifications	Low to moderate variability	Long procedure	High cost	Wide availability but limited use	High	Metallic implants, pacemaker, claustrophobia, kidney disease	[21]
Tomosynthesis	Uses X-rays to produce 3D images	Breast compression	Ionizing radiation	Optimized detection in dense tissues	Moderate variability	Fast procedure	Moderate cost	Wide availability	Moderate	Not recommended in young or pregnant women	[22]

N/A: Not applicable; 2D: Two dimensional; 3D: Three dimensional.

## 2. Materials and Methods

This prospective, transversal, and descriptive study was conducted at the Instituto Jalisciense de Cancerología (IJC); it involved all western regions of Mexico. This study was carried out following the regulations of the General Health Law on Health Research, the guidelines established by COFEPRIS, the Health Law of the State of Jalisco, the guidelines of the Ethics and Research Committee of the OPD of IJC (with registration number CONBIOETICA-14-CEI-004-20170421), and the guidelines established by the E6 Good Clinical Practice (GCP) Guide of the International Conference on Harmonization (ICH). All the patients gave written informed consent.

### 2.1. Patients

The data collection was from January 2017 to April 2023. The inclusion criteria were female patients aged 18 to 78 with a positive or negative solid lesion in the breast, determined through examination with B-mode US with SE and histological confirmation in all cases. One hundred and eight breast biopsies of patients were negative, and one hundred and eight were BC positive. All patients were paired according to age.

### 2.2. Ultrasound and Strain Elastography

**Figure 1:**
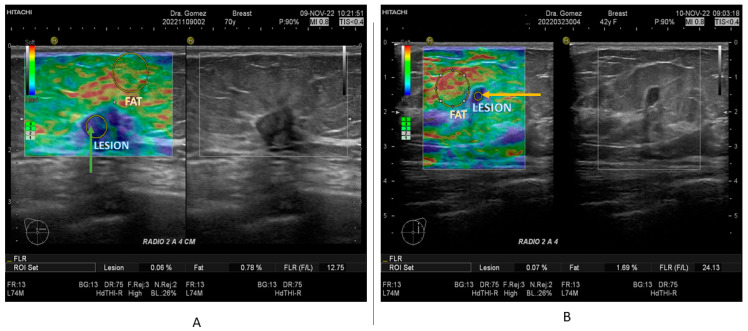
Images of B mode US and SE, corresponding to (**A**) TN tumor in a female of 70 years of age (green arrow) with a F/L index of 12.75; (**B**) Luminal A tumor in a female of 42 years of age (blue arrow) with a F/L index of 24.13.

**Figure 2:**
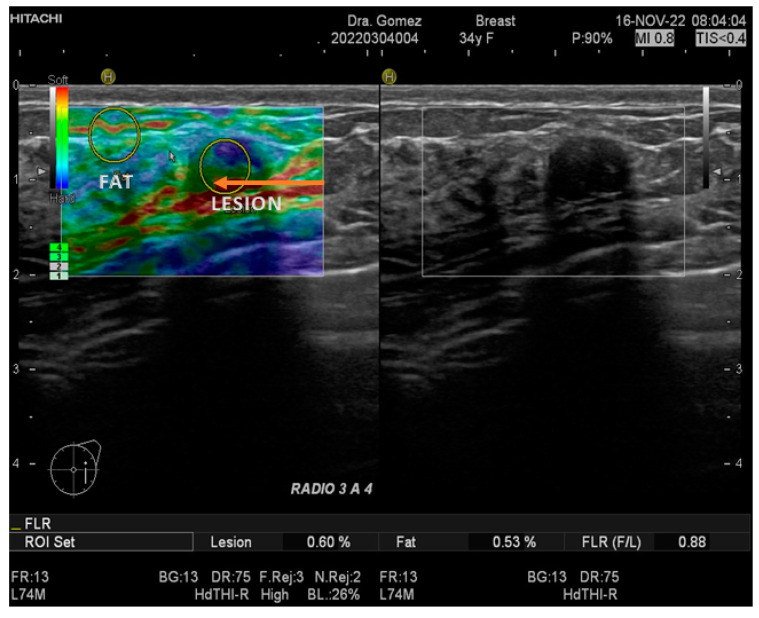
Images of B mode US and SE, corresponding to a negative biopsy in a female of 34 years of age (orange arrow) with a F/L index of 0.88.

In this study, all patients with any mass or suspicion of them underwent US exploration and SE, performed by three radiologists with more than 15 years of experience in breast imaging. All measurements and images were acquired with a Hitachi Avius (Hitachi Medical, Tokyo, Japan) equipped with a multifrequency linear transducer from 7 to 12 MHz, with color Doppler and elastography by setting the transducer to longitudinal scans. We used SE elastography in all patients, measuring three times with slight compression, in line with the World Federation for Ultrasound in Medicine & Biology (WFUMB) Guidelines, thereby obtaining a final average (Figure 1 and Figure 2) [23]. All lesions were classified by the 5th edition of Breast Imaging Reporting and Data System (BI-RADS), where category 2 is benign, category 3 is probably benign, category 4 is suspicion of malignancy and is segmented into 4A (low suspicious for malignancy), 4B (moderately suspicious for malignancy), and class 4C highly suspicious for malignancy (in all of them, biopsy is required), and category 5 is highly suspicious for malignancy (biopsy mandatory) [24].

Primary care clinics and BC units referred to the IJC were reclassified through new mammography, B-mode ultrasonography, and elastography. Of all the cases, 50.9% were recategorized into BI-RADS; the data collected for the analysis were the BI-RADS assigned by the IJC radiologists.

### 2.3. Histological and Immunohistochemistry Evaluation

After performing the elastography, all patients underwent a biopsy. The tissues were formalin-fixed, paraffin-embedded (FFPE), and stained with hematoxylin and eosin for the classification of tumors according to the WHO classification of breast tumors. To determine the histological features, the modified Scarf Bloom- Richardson (SBR) grading system was used. Immunohistochemistry (IHC), which serves to determine the molecular subtypes of BC, was assessed according to the 2011 St. Gallen Consensus Conference and the 13th St. Gallen International Breast Cancer Conference (Luminal A corresponding to ER+ and/or PR+, HER2−, and Ki-67 < 14%; Luminal B corresponding to ER+ or PR+/−, HER2− and Ki-67 > 14%; Luminal B-like corresponding to ER+, HER2+, any Ki-67, and any PR; HER2+ corresponding to ER/PR−; and triple-negative (TN), corresponding to ER/PR− and HER2−) [25,26]. Pathologists with over 20 years of experience in diagnosing breast tumors within the IJC examined all tissues under light microscopy.

### 2.4. Clinical Stages in BC

Patients who tested positive for BC in this study underwent surgical intervention. A clinical oncologist determined the clinical stage of the disease according to the Eighth Edition of the AJCC Cancer Staging Manual: Breast Cancer [27], in conjunction with a histopathological review of the histological grade and the molecular subtype of the BC. All clinical and surgical oncologists involved in this study had more than 10 years of experience.

### 2.5. Statistical Analysis

All data analyses and plots were performed using IBM Corp. (2017) SPSS Statistics for Windows, Version 25.0. Armonk, New York, NY, USA (https://www.ibm.com/mx-es/products/spss-statistics (accessed on 15 November 2023)), JASP Team (2023) JASP (version 0.18.1.0) (https://jasp-stats.org/ (accessed on 15 November 2023)), and GraphPad Prism version 9.02 for windows (https://www.graphpad.com/updates/prism-900-release-notes (accessed on 15 November 2023)). Quantitative data were represented as means and standard deviations; qualitative data were represented as frequencies or percentages. Normality was performed using the Shapiro-Wilk test.

Inferential analyses were made with the Student’s *t*-test for normal distribution and the Mann’s U-test and Kruskal Wallis test for non-normal distribution. Spearman’s Rho correlation was applied. Chi-square or Fisher’s exact test was used for qualitative data. Principal Component Analysis (PCA) was used to summarize features. Subsequently, significant variables (*p* < 0.05) were selected and included in a multivariable logistic regression model and neural network analysis, which were evaluated using the ROC curve and the Akaike criterion. The Youden index was used to identify the cut-off value for the F/L index.

For the neural network analysis settings, we implemented a multilayer perceptron neural network with a fixed seed of 2 million, 2000 epochs, and two hidden layers. The activation functions for the hidden layer and output layer used the hyperbolic tangent.

## 3. Results

A total of 216 patients were included in this study. The mean age of women with malignant biopsies was 51.38 ± 10.80 years; in contrast, the mean age of patients with benign biopsies was 50.64 ± 10.81 years. The average tumor diameter in patients with negative biopsies was 16.88 ± 12.86 mm, compared to patients with positive biopsies, which was 24.20 ± 17.67 mm. The F/L index mean value was 3.70 ± 2.57 for benign lesions; meanwhile, for malignant lesions, the average value for the F/L index was 18.10 ± 17.01 (Table 2).

### 3.1. Principal Component Analysis

**Figure 3:**
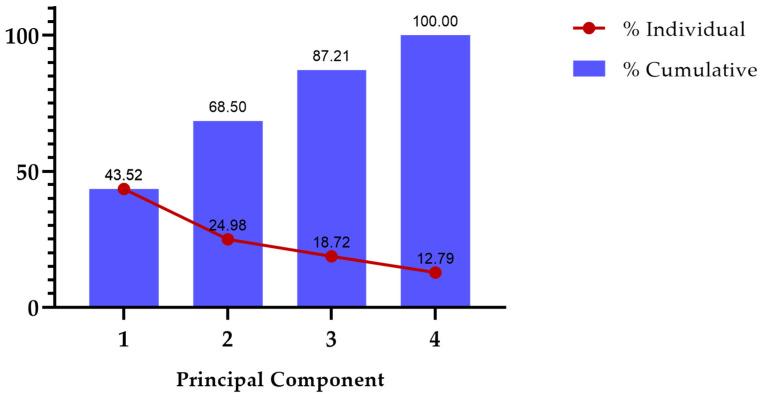
Proportion of variance. Independent variables: age, tumor diameter, F/L index, and BI-RADS classification. Biopsy outcome was considered as a dependent variable.

**Figure 4:**
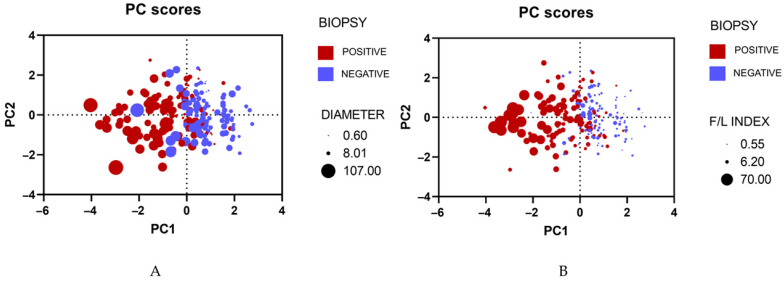
(**A**) Principal Component Analysis of biopsies and tumor diameter; (**B**) Principal Component Analysis of biopsies and F/L index.

Principal Component Analysis (PCA) was carried out to visualize and explain our main clinical features. Age, tumor diameter, F/L index, and BI-RADS classification as independent variables and biopsy outcome as a dependent variable were incorporated into the PCA. After the analysis, there were two principal components (PCs) which explained 68.5% of the variance (Figure 3). Both graphics manifested similar outcomes; patients who tested positive for malignancy tended to concentrate on the left quadrant, while patients who tested negative for malignancy focused on the right quadrant. On top of that, a higher F/L index and a larger tumor diameter were prevalent in the left quadrant of both PCAs (Figure 4).

### 3.2. Histological Type, BI-RADS Assignment

Among all the patients found to be positive for malignancy, 94 were diagnosed with invasive ductal carcinoma (IDC) (87.04%) and 10 with invasive lobular carcinoma (ILC) (9.26%). On the other hand, for benign tumors, fibroadenoma was predominant in 59 patients (54.63%), followed by fibrocystic changes in 9 patients (8.33%), ductal hyperplasia in 15 patients (13.89%), lobular hyperplasia in 1 patient (0.93%), and atypia in 2 patients (1.85%). Mastitis, lipomas, fat necrosis, and adenosis were categorized as “Other benign changes” (20.37%). Regarding BI-RADS classification, we observed a higher proportion of BI-RADS 5 (30.09%), followed by BI-RADS 4A (23.15%), BI-RADS 4C (14.81%), BI-RADS 4B (13.89%), and BI-RADS 3 (13.43%). BI-RADS 2 and BI-RADS 6 were less prevalent, accounting for 2.31% of all the cases (Table 3).

### 3.3. Clinical Stages, Grading Tumors, and Molecular Subtype BC

Clinical stage III (38.89%) was the most common, followed by stage II (34.26%), stage I (14.81%), and stage IV (9.26%). Moreover, the proportion of histological grade was as follows: grade 1 or well-differentiated (1.85%), grade 2 or moderately differentiated (70.37%), and grade 3 or poorly differentiated (19.44%). Concerning molecular subtypes, we observed Luminal B (35.19%) and Luminal A (33.33%) in higher proportions, followed by TN (15.74%), and HER2-enriched (11.11%) (Table 4). We performed a Chi square test with non-significant results to compare clinical stages and molecular subtypes.

**Figure 5:**
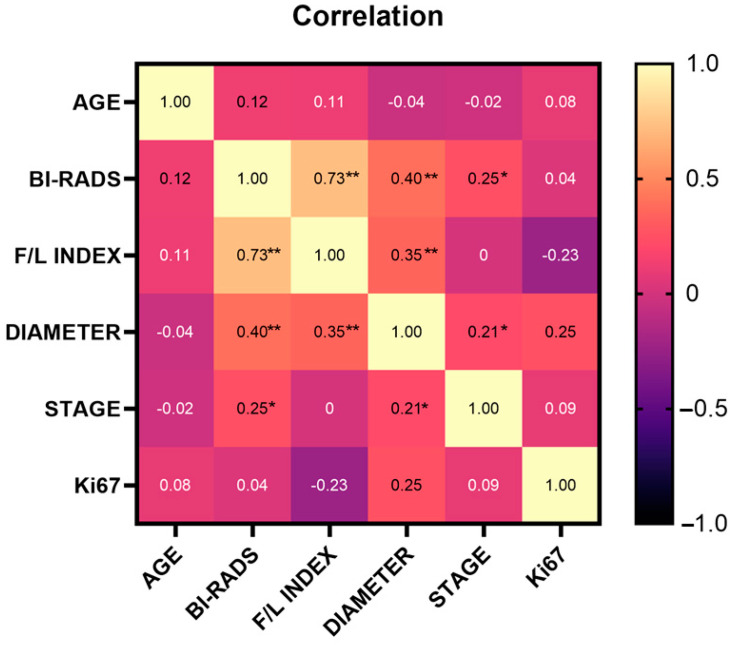
Spearman’s Correlation matrix of variables. The strongest correlations are marked with ** for significant correlation, i.e., *p* < 0.01 (bilateral), or * for significant correlation, i.e., *p* < 0.05 (bilateral).

Our results demonstrated that the F/L index had a positive and significant correlation between BI-RADS classification (r = 0.73) and tumor diameter (r = 0.35); similarly, BI-RADS classification was positively and significantly correlated with tumor diameter (r = 0.40) and clinical stage (r = 0.25). At the same time, clinical stage and tumor diameter showed a positive and significant correlation (r = 0.21) (Figure 5).

**Figure 6:**
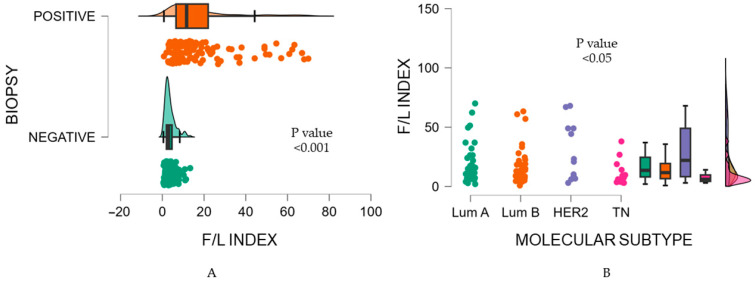
(**A**) Biopsy and F/L index; (**B**) Molecular subtype and F/L index.

Furthermore, we found a significant association between the F/L index and the tumor biopsy (*p* < 0.001). A lower F/L index was identified in malignancy-negative biopsies and a higher F/L index in malignancy-positive biopsies (3.70 vs. 18.10) (Figure 6).

Consequently, the Kruskal-Wallis test showed that the F/L index presented significantly different values according to molecular subtype classification (*p* = 0.02) (Figure 4B). HER2-enriched showed a F/L index of 29.66 ± 24.42, contrary to TN, with an average F/L index of 10.15 ± 9.52. Luminal subtypes revealed similar values, with a mean F/L index of 19.34 ± 16.73 for Luminal A and a mean F/L index of 16.53 ± 15.35 for Luminal B (Table 5). Post hoc tests indicated a significant difference between Luminal A vs. TN and HER2-enriched vs TN (*p* < 0.05) (Table 6).

### 3.4. Binary Logistic Regression and Neural Network Performance for BC

**Figure 7:**
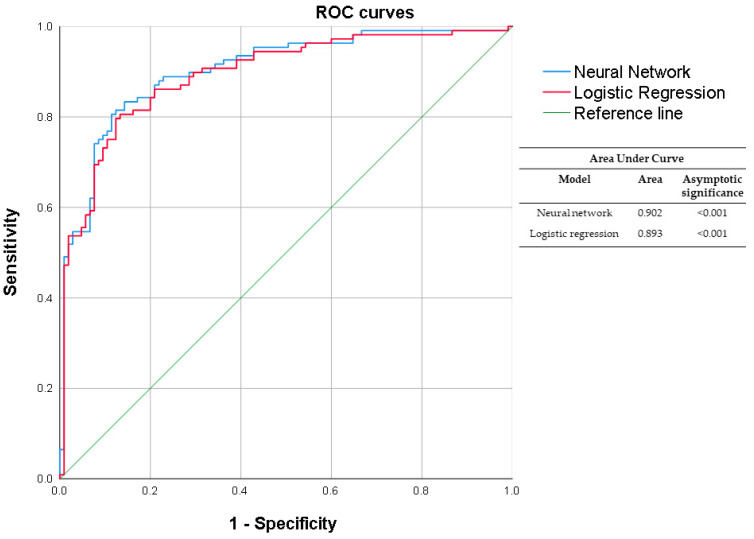
ROC curve comparing neural network versus logistic regression. The neural network performs 0.902 of AUC with a *p* < 0.001; Logistic regression shows an AUC of 0.893 and a *p* < 0.001.

Our binary logistic regression and neural network analysis included age, tumor diameter, and F/L index as independent variables in the model. In our logistic regression, a minor standard error was observed. Additionally, the F/L index was the main predictive factor to determine malignancy in biopsy, with an odds ratio (OR) of 1.48. Age was also significant, with a 5% reduction in risk (OR 0.95). Tumor diameter was not a significant factor when controlling variables with the Wald test (Table 7). Regarding our binary logistic regression analysis, we developed a receiver operating characteristic curve (ROC) and obtained an Area Under the Curve (AUC) of 0.893, a sensitivity of 79.63%, a specificity of 87.62%, a positive predictive value (+PV) of 86.9%, and a negative predictive value (−PV) of 80.7%. Our neural network analysis had similar outcomes, with an AUC value of 0.902, a sensitivity of 80.56%, a specificity of 88.57%, a +PV of 87.9% and a −PV of 81.6%. Both results were significant, with a *p* < 0.001. The normalized importance of the independent variables was calculated as follows: age at 2.4%, tumor diameter at 27.9%, and F/L index at 100%. Further, the cut-off value for the F/L index was >5.7, with a sensitivity and specificity of 84.26% (Figure 7).

## 4. Discussion

This manuscript reports the relationship between the F/L index, molecular subtypes of BC, and clinical stages of BC. An essential element distinguishing this work from previous publications on SE was that the patients were matched by age.

A recent publication by Zhu et al. [28] utilized B-mode ultrasound, real-time strain elastography (RTE), color Doppler flow imaging (CDFI), and contrast-enhanced ultrasound (CEUS) techniques in 85 patients with histologically confirmed BC and defined molecular subtypes. Ultrasound RTE and CDFI were significant in our binary logistic regression analysis (*p* < 0.001 and *p* = 0.036, respectively) for predicting the Luminal A molecular subtype of BC. For Luminal B, ultrasound RTE and CEUS showed *p* = 0.016 and *p* = 0.036, respectively. Meanwhile, only CEUS discriminated HER2-enriched from other subtypes (p = 0.039). For the triple negative BC subtype (TNBCs), only CDFI (p = 0.002) obtained significant differences compared to non-TNBCs. The authors conclude that ultrasound RTE is affordable for Luminal subtypes, while CDFI and CEUS techniques are beneficial for measuring hypovascularity and hypervascularity, particularly in TNBCs and Luminal B breast tumors. In this sense, our findings agree with those of Zhu et al. in terms of the significant difference between the various molecular subtypes. Our research agrees that TNBCs were the softest tumors. Regarding Luminal tumors, Luminal A had the highest stiffness, as observed in our study. However, in our research, the HER2-enriched subtype was the hardest tumor, in contrast to results published by Zhu et al., who cited Luminal A as the hardest tumor.

On the other hand, Hayashi et al. in 2015 [29] conducted an observational, retrospective study involving 503 patients with invasive BC. They considered clinicopathological features like molecular subtype, tumor size, invasive tumor size, lymph node metastasis, nuclear grade, age, menopausal status, BMI, and breast density according to BI-RADS. They studied a subsample of 164 patients with frozen tissue and determined stroma-related gene expressions. They found that clinical tumor stiffness correlated with lymph node involvement (*p* = 0.0005) and invasive tumor size (*p* < 0.0001). Multivariable analysis showed that the stiffness of the primary breast tumor correlated with axillary lymph node metastasis as an independent factor. Regarding gene expression, they reported higher expression of lysyl oxidase in hard tumors with FDR-adjusted *p* = 0.0279, suggesting that the extracellular matrix plays a vital role in the etiology of tumor stiffness. Although we did not study the same variables as Hayashi et al., we obtained a similar correlation between tumor size and the F/L index, meaning larger size correlated with greater hardness of the tumor (r = 0.35, *p* < 0.001) when all tumors were analyzed together, regardless of molecular subtype.

In 2017, Jin et al. [30] investigated SE and its relationship with tumor pathology and tumor stiffness, as determined by the elasticity score and stiffness index. Jin et al. included 291 patients with invasive BC. They found that 79% of tumors had a high stiffness index, with an average hardness percentage of 82.32 ± 15.72%. A statistical analysis found a relationship between hardness with histological grade and molecular subtypes, i.e., grade I-II tumors were harder than grade III tumors, and Luminal A tumors were harder than Luminal B, HER2-enriched, and TNBC tumors. There was no relationship with histologic lineage or type. Compared to our findings, we similarly found that Luminal A was one of the subtypes with high hardness, but in our case, HER2-enriched had higher hardness than Luminal A. This may be because few HER2-enriched tumors were analyzed in both studies and the populations differed, leading to varied results. However, unlike Jin et al., we did not confirm a relationship between hardness and degree of differentiation in our analysis. This may be because we only included two cases with grade I, which could reduce the power to detect an association with this variable.

One of the most extensive studies about elastography and the F/L index is that of Togawa et al. [31]. That research group studied 1288 women with BI-RADS 3 and 4a–4c lesions through B-mode ultrasound, evaluating SWE with the F/L index. F/L values were contrasted with biopsy results. They found 368 (28.6%) malignant tumors. After evaluation with conventional B-mode ultrasound, 53.8% of benign lesions by biopsy were classified as BI-RADS 4, corresponding to false positives. In contrast, after ultrasound evaluation, only 1.39% were classified as BI-RADS 3 but were positive after biopsy (false negatives). Our findings showed similar results; however, 66.66% of benign biopsies at our site were classified as BI-RADS 4a–4c (false positives). We hypothesize that this divergence could be because Togawa et al. used SWE, different from our study. Also, the populations were ethnically diverse, and breast tissue composition differs between Caucasian and Asian populations. Notably, the Mexican population has significant Asian ancestry [32,33].

Last year, Lee et al. [34] investigated how fibrotic focus (FF) affects strain SE in 151 patients with BC. Among all patients, 46.9% were FF-positive; nevertheless, SE did not show significant differences between groups (*p* = 0.633). The rest of their results align with previous reports. They found significant differences in a univariate analysis of clinical characteristics between three groups classified by SE index (positive vs. equivocal vs. negative). Positive SE index patients were older (*p* = 0.044), had larger tumors (*p* = 0.004), and were in stage II compared to other clinical stages (*p* = 0.028). This study demonstrated that clinicopathological characteristics correlate with tumor stiffness and poor prognostic features of BC. In our research, we did not evaluate fibrotic focus. However, similar to Lee et al., we found a correlation between F/L index with age and tumor diameter (*p* < 0.0001), as well as clinical BC stage and BI-RADS via Spearman’s correlation coefficient r = 0.25. Notably, we included BC stage IV because, in Mexico, sociodemographic factors impact stage at diagnosis, with most patients unfortunately being diagnosed in the late stages (stage III and IV).

Similarly, Çorapli et al. [35], aimed to establish a correlation between SE and molecular subtypes of BC in 195 patients. However, they did not find significant results and concluded that SE is ineffective at identifying BC molecular subtypes. In contrast, our results showed significance between Luminal A vs. TN, with *p* < 0.005, and HER2-enriched vs. TN, with *p* < 0.004, possibly due to our paired patient analysis.

In 2022, Shehata et al. [36] evaluated the performance of ultrasound elastography score (ES), quantitative mass strain ratio (SR), and shear wave elasticity ratio (SWE) in discerning benign from malignant breast tumors. The study enrolled 51 patients with 77 histological breast masses, of which 57 tested positive and 20 negative for malignancy. For SR, they reported a statistical difference between positive and negative biopsies (*p* < 0.001). Furthermore, the cut-off value set for SR was >4.6 with 96.5% sensitivity and 80% specificity. These results align with those of our study. Shehata emphasized that semi-quantitative elastography assessed by the F/L index is more objective than other stiffness measurement methodologies, as it considers the patient’s tissue as a reference. Thus, a relative reference to non-tumor tissue is essential for adequate predictions.

A systematic review and meta-analysis by Mutala et al. [37] reported a sensitivity and specificity of 0.86 and 0.74, respectively, for a cut-off point of 2.81. However, they cautioned about the importance of proper imaging tool function and considering the manufacturer model when using sonoelastography and strain ratio values for lesion characterization.

Finally, in our research, we observed some limitations. Constructing reference tables by age group and other characteristics will be required to establish the optimal cut-off point. For this purpose, larger samples and precise clinical features are needed.

## 5. Conclusions

Our study demonstrated that SE and F/L index are associated with mammography, BI-RADS classification, biopsy, and molecular subtype in BC. SE has a good predictive ability, is a fast procedure, and is minimally invasive, which indicates its usefulness for BC diagnosis, particularly when mammography is not adequate according to the tissue characteristics. Binary logistic regression and neural network suggested a high predictive value and supported the clinical use of SE as a complement in the diagnosis of BC.

## Figures and Tables

**Table 2:** Descriptives for age, tumor diameter, F/L index, and normality test.

Descriptives Statistics	Age		Tumor Diameter		F/L Index	
Biopsy result	Neg	Pos	Neg	Pos	Neg	Pos
Median	49.50	50.00	14.00	24.00	2.96	11.66
Mean	50.64	51.38	16.88	24.20	3.70	18.10
SD	10.81	10.80	12.86	17.67	2.57	17.01
*p*-value of Shapiro-Wilk	0.18	0.61	<0.001 *	<0.001 *	<0.001 *	<0.001 *

SD: Standard Deviation; Neg: Negative; Pos: positive; Shapiro-Wilk test for normality; * *p*-value significance < 0.05.

**Table 3:** Frequency of clinical features.

Malignant Tumors	n	Percent (%)
IDC	94	87.04%
ILC	10	9.26%
Missing	4	3.70%
Total	108	100.00%
**Benign tumors**	**n**	**Percent (%)**
Fibroadenoma	59	54.63%
Fibrocystic	9	8.33%
Ductal Hyperplasia	15	13.89%
Lobular Hyperplasia	1	0.93%
Atypia	2	1.85%
Other benign changes	22	20.37%
Total	108	100.00%
**BI-RADS**	**n**	**Percent (%)**
2	5	2.31%
3	29	13.43%
4A	50	23.15%
4B	30	13.89%
4C	32	14.81%
5	65	30.09%
6	5	2.31%
Total	216	100.00%

IDC: Invasive ductal carcinoma, ILC: Invasive lobular carcinoma, BI-RADS: Breast Imaging Reporting and Data System.

**Table 4:** Clinical features of malignancy tumors.

Stage	n	Percent (%)
I	16	14.81%
II	37	34.26%
III	42	38.89%
IV	10	9.26%
Not reported	3	2.78%
Total	108	100.00%
**Grade**	**n**	**Percent (%)**
1	2	1.85%
2	76	70.37%
3	21	19.44%
Not reported	9	8.33%
Total	108	100.00%
**Molecular subtype**	**n**	**Percent (%)**
HER2-enriched	12	11.11%
Luminal A	36	33.33%
Luminal B	38	35.19%
TN	17	15.74%
Not reported	5	4.63%
Total	108	100.00%

TN: Triple Negative, HER2: human epidermal growth factor receptor 2.

**Table 5:** Descriptives for F/L INDEX by subtype.

Subtype	n	Mean	Median	SD	SE	Coefficient of Variation
Luminal A	36	19.34	13.60	16.73	2.79	0.87
Luminal B	38	16.53	11.70	15.35	2.49	0.93
HER2-enriched	12	29.66	22.00	24.42	7.05	0.82
TN	17	10.15	6.08	9.52	2.31	0.94

SD: Standard deviation; SE: Standard error; TN: Triple negative; HER2: human epidermal growth factor receptor 2.

**Table 6:** Post hoc comparison with Kruskal-Wallis test.

Intra-Group Comparison	Uncorrected *p*-Value	Bonferroni *p* Value	FDR *p*-Value
Luminal A—Luminal B	0.38	1.00	0.48
Luminal A—HER2-enriched	0.48	1.00	0.48
Luminal A—TN	0.007	0.048 *	0.024 *
Luminal B—HER2-enriched	0.18	1.00	0.36
Luminal B—TN	0.05	0.288	0.48
HER2-enriched–TN	0.006	0.041 *	0.024 *

TN: Triple negative; HER2: human epidermal growth factor receptor 2; FDR: False discovery rate. Note: Dunn’s post hoc test was performed. All values have been adjusted by Bonferroni correction and Benjamini-Hochberg (FDR) for multiple comparisons. * *p* < 0.05.

**Table 7:** Binary Logistic Regression Coefficients.

			Wald Test	95% CI	
	Standard Error	Odds Ratio	*p*	Lower Bound	Upper Bound
Age	0.01	0.95	<0.001	−0.07	−0.04
F/L index	0.06	1.48	<0.001	0.27	0.52
Tumor diameter	0.01	1.01	0.62	−0.02	0.03

Note. Biopsy level ‘POSITIVE’ is coded as class 1. CI: Confidence interval, df: degrees of freedom.

## Data Availability

The raw data supporting the conclusions of this article will be made available by the corresponding authors on request.

## References

[1] World Health Organization Global Cancer Observatory Cancer Today. https://gco.iarc.fr/today/en.

[2] Iranmakani S., Mortezazadeh T., Sajadian F., Ghaziani M.F., Ghafari A., Khezerloo D., Musa A.E. (2020). A Review of Various Modalities in Breast Imaging: Technical Aspects and Clinical Outcomes. Egypt. J. Radiol. Nucl. Med..

[3] Ormachea J., Parker K.J. (2020). Elastography Imaging: The 30 Year Perspective. Phys. Med. Biol..

[4] Doyley M.M., Parker K.J. (2014). Elastography: General Principles and Clincial Applications. Ultrasound Clin..

[5] Sigrist R.M.S., Liau J., Kaffas A.E., Chammas M.C., Willmann J.K. (2017). Ultrasound Elastography: Review of Techniques and Clinical Applications. Theranostics.

[6] Chee C., Lombardo P., Schneider M., Danovani R. (2019). Comparison of the Fat-to-Lesion Strain Ratio and the Gland-to-Lesion Strain Ratio With Controlled Precompression in Characterizing Indeterminate and Suspicious Breast Lesions on Ultrasound Imaging. J. Ultrasound Med..

[7] Zhi H., Xiao X.-Y., Yang H.-Y., Wen Y.-L., Ou B., Luo B.-M., Liang B. (2008). Semi-Quantitating Stiffness of Breast Solid Lesions in Ultrasonic Elastography. Acad. Radiol..

[8] Franco Uliaque C., Pardo Berdún F.J., Laborda Herrero R., Pérez Lórenz C. (2016). Utilidad de la elastografía semicuantitativa para predecir la malignidad de los nódulos tiroideos. Radiología.

[9] Barr R.G. (2019). Future of Breast Elastography. Ultrasonography.

[10] Youk J.H., Son E.J., Gweon H.M., Han K.H., Kim J.-A. (2015). Quantitative Lesion-to-Fat Elasticity Ratio Measured by Shear-Wave Elastography for Breast Mass: Which Area Should Be Selected as the Fat Reference?. PLoS ONE.

[11] Seo M., Ahn H.S., Park S.H., Lee J.B., Choi B.I., Sohn Y., Shin S.Y. (2018). Comparison and Combination of Strain and Shear Wave Elastography of Breast Masses for Differentiation of Benign and Malignant Lesions by Quantitative Assessment: Preliminary Study. J. Ultrasound Med..

[12] Sandrin L., Fourquet B., Hasquenoph J.-M., Yon S., Fournier C., Mal F., Christidis C., Ziol M., Poulet B., Kazemi F. (2003). Transient Elastography: A New Noninvasive Method for Assessment of Hepatic Fibrosis. Ultrasound Med. Biol..

[13] Fang C., Huang D.Y., Sidhu P.S. (2019). Elastography of Focal Testicular Lesions: Current Concepts and Utility. Ultrasonography.

[14] Zhao C.-K., Xu H.-X. (2019). Ultrasound Elastography of the Thyroid: Principles and Current Status. Ultrasonography.

[15] Yılmaz E., Yılmaz A., Aslan A., Inan I., Evren M.C., Tekesin K. (2017). Real-Time Elastography for Differentiation of Breast Lesions. Pol. J. Radiol..

[16] ElMowalled S. (2023). Value of Ultrasound Elastography in Combined with Mammography in Evaluation of Indeterminate Breast Lesions. Benha J. Appl. Sci..

[17] D’Orsi C., Sickles E., Mendelson E., Morris E. (2013). ACR BI-RADS® Ultrasound. ACR BI-RADS® Atlas, Breast Imaging Reporting and Data System.

[18] You Y., Song Y., Li S., Ma Z., Bo H. (2019). Quantitative and Qualitative Evaluation of Breast Cancer Prognosis: A Sonographic Elastography Study. Med. Sci. Monit..

[19] Farrokh A., Wojcinski S., Degenhardt F. (2010). Diagnostische Aussagekraft der Strain-Ratio-Messung zur Unterscheidung zwischen malignen und benignen Brusttumoren. Ultraschall Med..

[20] Heywang-Köbrunner S.H., Hacker A., Sedlacek S. (2011). Advantages and Disadvantages of Mammography Screening. Breast Care.

[21] Radhakrishna S., Agarwal S., Parikh P.M., Kaur K., Panwar S., Sharma S., Dey A., Saxena K.K., Chandra M., Sud S. (2018). Role of Magnetic Resonance Imaging in Breast Cancer Management. South Asian J. Cancer.

[22] Kleinknecht J.H., Ciurea A.I., Ciortea C.A. (2020). Pros and Cons for Breast Cancer Screening with Tomosynthesis—A Review of the literature. Med. Pharm. Rep..

[23] Barr R.G., Nakashima K., Amy D., Cosgrove D., Farrokh A., Schafer F., Bamber J.C., Castera L., Choi B.I., Chou Y.-H. (2015). WFUMB Guidelines and Recommendations for Clinical Use of Ultrasound Elastography: Part 2: Breast. Ultrasound Med. Biol..

[24] Niknejad M., Weerakkody Y. (2010). Breast Imaging-Reporting and Data System (BI-RADS). Radiopaedia.org.

[25] Gnant M., Harbeck N., Thomssen C. (2011). St. Gallen 2011: Summary of the Consensus Discussion. Breast Care.

[26] Thomssen C., Balic M., Harbeck N., Gnant M. (2021). St. Gallen/Vienna 2021: A Brief Summary of the Consensus Discussion on Customizing Therapies for Women with Early Breast Cancer. Breast Care.

[27] Giuliano A.E., Edge S.B., Hortobagyi G.N. (2018). Eighth Edition of the AJCC Cancer Staging Manual: Breast Cancer. Ann. Surg. Oncol..

[28] Zhu J.-Y., He H.-L., Jiang X.-C., Bao H.-W., Chen F. (2023). Multimodal Ultrasound Features of Breast Cancers: Correlation with Molecular Subtypes. BMC Med. Imaging.

[29] Hayashi M., Yamamoto Y., Sueta A., Tomiguchi M., Yamamoto-Ibusuki M., Kawasoe T., Hamada A., Iwase H. (2015). Associations Between Elastography Findings and Clinicopathological Factors in Breast Cancer. Medicine.

[30] Jin Y., Fenghua L., Jing D., Yifen G. (2017). Strain Elastography Features in Invasive Breast Cancer: Relationship between Stiffness and Pathological Factors. Int. J. Clin. Exp. Med..

[31] Togawa R., Pfob A., Büsch C., Alwafai Z., Balleyguier C., Clevert D., Duda V., Fastner S., Goncalo M., Gomez C. (2023). Potential of Lesion-to-Fat Elasticity Ratio Measured by Shear Wave Elastography to Reduce Benign Biopsies in BI-RADS 4 Breast Lesions. J. Ultrasound Med..

[32] Patel B.K., Pepin K., Brandt K.R., Mazza G.L., Pockaj B.A., Chen J., Zhou Y., Northfelt D.W., Anderson K., Kling J.M. (2022). Association of Breast Cancer Risk, Density, and Stiffness: Global Tissue Stiffness on Breast MR Elastography (MRE). Breast Cancer Res. Treat..

[33] Rodríguez-Rodríguez J.E., Ioannidis A.G., Medina-Muñoz S.G., Barberena-Jonas C., Blanco-Portillo J., Quinto-Cortés C.D., Moreno-Estrada A. (2022). The Genetic Legacy of the Manila Galleon Trade in Mexico. Philos. Trans. R. Soc. B.

[34] Lee N.-R., Oh H.-K., Jeong Y.-J. (2022). Clinical Significance of Ultrasound Elastography and Fibrotic Focus and Their Association in Breast Cancer. J. Clin. Med..

[35] Çorapli M., Bulut H.T., Örmecï A.G., Alakuş H. (2022). Relationship between Strain Elastography and Histopathological Parameters in Breast Cancer. Cukurova Med. J..

[36] Shehata R.M.A., El-Sharkawy M.A.M., Mahmoud O.M., Kamel H.M. (2022). Qualitative and Quantitative Strain and Shear Wave Elastography Paradigm in Differentiation of Breast Lesions. Egypt. J. Radiol. Nucl. Med..

[37] Mutala T.M., Mwango G.N., Aywak A., Cioni D., Neri E. (2022). Determining the Elastography Strain Ratio Cut off Value for Differentiating Benign from Malignant Breast Lesions: Systematic Review and Meta-Analysis. Cancer Imaging.

